# Integrating MaxEnt and Random Forest Models to Assess Habitat Suitability of Black‐Necked Cranes, A Case Study in Nyingchi City

**DOI:** 10.1002/ece3.72058

**Published:** 2025-09-07

**Authors:** Jiujiu Wu, Min Zheng, Zhongbin Wang

**Affiliations:** ^1^ Tibet Agriculture and Animal Husbandry College Nyingchi China

**Keywords:** black‐necked crane, habitat suitability modeling, MaxEnt, Random Forest models

## Abstract

Understanding the spatial distribution of rare species is fundamental to biodiversity conservation. The black‐necked crane ( *Grus nigricollis* ), a flagship species of alpine wetlands and a first‐class nationally protected species in China, serves as an important indicator for ecosystem health. Based on the had data and ecological environment data, this study used the Maximum Entropy model (MaxEnt) and Random Forest model (RF) to predict the suitable distribution area of the black‐necked crane. The Random Forest model exhibited high predictive accuracy, with an AUC of 0.945, closely aligning with known crane distribution patterns. Key environmental determinants of habitat suitability were identified as distance to buildings (d_b), distance to roads (d_r), and isothermality (Bio3), with average contribution rates of 15.1%, 15.05%, and 5.85%, respectively. High‐probability suitable areas were primarily concentrated in riparian wetlands of Nyingchi City, with an optimal habitat core at the T‐shaped valley confluence of the Yarlung Tsangpo and Nyang rivers. Through comparative analysis of MaxEnt and RF, this study significantly reduced spatial uncertainties in habitat suitability predictions. These findings provide critical spatial baselines for targeted conservation strategies of this sacred plateau species, particularly in maintaining ecological connectivity under climate change scenarios.

## Graphical abstract

In this study, based on the distribution location data and ecological environment data of black‐necked cranes, the maximum entropy model (MaxEnt) and random forest model (RF) were used to predict the suitable distribution area of black‐necked cranes.

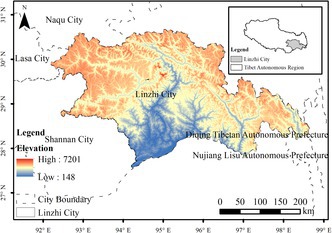

## 1 Introduction

Biodiversity, encompassing the diversity of all life forms on Earth, forms the foundation for human survival and development. Since the 21st century, humans have had a more profound impact on the changes to modify natural ecosystems, which has led to a gradual biodiversity decline, driven primarily by severe habitat destruction and fragmentation (Pereira et al. 2012). Against this backdrop, understanding species distribution patterns and habitat suitability has become increasingly critical. Habitat suitability assessment serves as a cornerstone for species‐specific conservation strategies (Larson et al. 2004; Fischer and Lindenmayer 2007; Kaminski and Elmberg 2014; Tellería 2016) and a key indicator of habitat quality (Grinnell 1917; OuYang et al. 2001). The importance of habitat suitability is that it can directly affect the population dynamics of species (such as birth, death, migration) and ecological adaptability (i.e., their ability to survive and reproduce). However, such assessments face inherent challenges, including species‐specific habitat selection across spatial hierarchies (Tellería 2016; Elafri et al. 2017) and the need to align with ecological structures and processes operating at multiple spatial and temporal scales (Nash et al. 2014). Consequently, evaluating wildlife habitat suitability has emerged as a global research priority for understanding shifts in species distributions (Gurnell et al. 2002; Peterson 2006; Hooper et al. 2012; Qiao et al. 2013). To address these challenges, elucidating habitat quality for focal species is essential. Generally speaking, when carrying out bird habitat suitability evaluation, the influence of vegetation type is more significant, and the main environmental variables need to be determined according to breeding and overwintering habits. The black‐necked crane is no exception. By identifying and analyzing environmental drivers of distribution, this approach facilitates the mapping of potential geographic ranges and establishes scientifically robust frameworks for conservation planning and protected area management (Hou et al. 2021).

Black‐necked crane ( *Grus nigricollis* ) is a large wading bird belonging to Gruiformes and Gruidae. It is listed as Near Threatened (NT) by IUCN and is the only crane species among the 15 extant members of the family Gruidae that exclusively inhabits high‐altitude plateau ecosystems throughout its life cycle (Wang et al. 1998; BirdLife International 2022). Globally, the population currently exceeds 17,000 individuals (Chen, Pu, Huang, et al. 2023b, 2023a), showing a steady recovery trend in recent decades. However, this species faces escalating threats from wetland habitat loss and climate change‐induced degradation of shallow palustrine wetlands (Li 2002). Abundant research has investigated the population abundance, distribution patterns, and habitat selection of the black‐necked crane. For instance, Meine conducted research on population abundance (Meine and Archibald 1996). Kong et al. highlighted the impacts of tourism activities, particularly predation risks and anthropogenic disturbances, emphasizing the necessity of maintaining safe distances between tourists and cranes (Kong et al. 2021). In the Napahai Provincial Nature Reserve of Yunnan, studies identified food availability, water resources, and human disturbance as critical environmental variables influencing habitat quality (Wang 2015). Furthermore, systematic monitoring programs have been established across the Tibetan Plateau, focusing on the species' wintering behaviors, including clustered foraging patterns.

Species distribution modeling (SDM) is a valuable tool for studying habitat suitability, identifying potential species distribution ranges, revealing key influencing factors, and providing critical scientific foundations for biodiversity conservation (Phillips et al. 2006; Elith and Leathwick 2009). In recent years, extensive research has been conducted on SDMs by scholars (Norberg et al. 2019). Depending on whether the indicator of species occurrence records is needed when building a model, habitat suitability models can be categorized into three types: statistical models, mechanistic models, and ecological niche models (Qiao et al. 2013; Elith and Leathwick 2009; Norberg et al. 2019; Liu et al. 2010, 2013; Bai et al. 2022). Widely used approaches include the Maximum Entropy model (MaxEnt) (Phillips et al. 2006; Elith et al. 2006), Random Forest model (RF) (Breiman 2001), Ecological Niche Factor Analysis (ENFA) (Hirzel et al. 2002), Generalized Linear Models (GLM) (Guisan et al. 2002), and Artificial Neural Networks (ANN) (Olden and Jackson 2002). Among these, MaxEnt demonstrates broad applicability and reduced bias by integrating species occurrence records with environmental variables. Its advantage lies in the ability to achieve accurate prediction results using only a small number of occurrence records, consistently yielding accurate predictions (Phillips and Dudík 2008; Merow et al. 2013; Radosavljevic and Anderson 2014). RF, as one of the most widely used machine learning algorithms, exhibits robustness to multicollinearity and outlier tolerance, making it versatile across disciplines (Sun et al. 2021). Although many studies have applied MaxEnt and RF separately, research that employs both models simultaneously is relatively rare. Single‐model predictions inherently carry uncertainties; even statistically validated results may deviate substantially from actual species distributions. Multi‐model comparisons effectively mitigate spatial uncertainties and optimize regional suitability assessments.

The black‐necked crane winters in the Tibetan Plateau and other regions. Nyingchi City is also one of its important wintering areas, providing essential resources such as food and habitats for the cranes to spend the winter. Most of the area of Nyingchi is made up of mountains and valleys. However, wintering black‐necked cranes in Nyingchi City exhibit spatially fragmented distributions, complicating conservation efforts. Therefore, to enhance the accuracy of habitat suitability predictions for black‐necked cranes in Nyingchi City and improve conservation outcomes for their spatially dispersed populations, this study employed a comparative modeling approach integrating MaxEnt and RF (Chen, Pu, Huang, et al. 2023b, 2023a). This framework effectively reduces spatial uncertainties, mitigates prediction biases associated with single‐model approaches, and identifies optimal habitat suitability zones for targeted conservation planning.

## 2 Materials and Methods

### 2.1 Study Area

**FIGURE 1:**
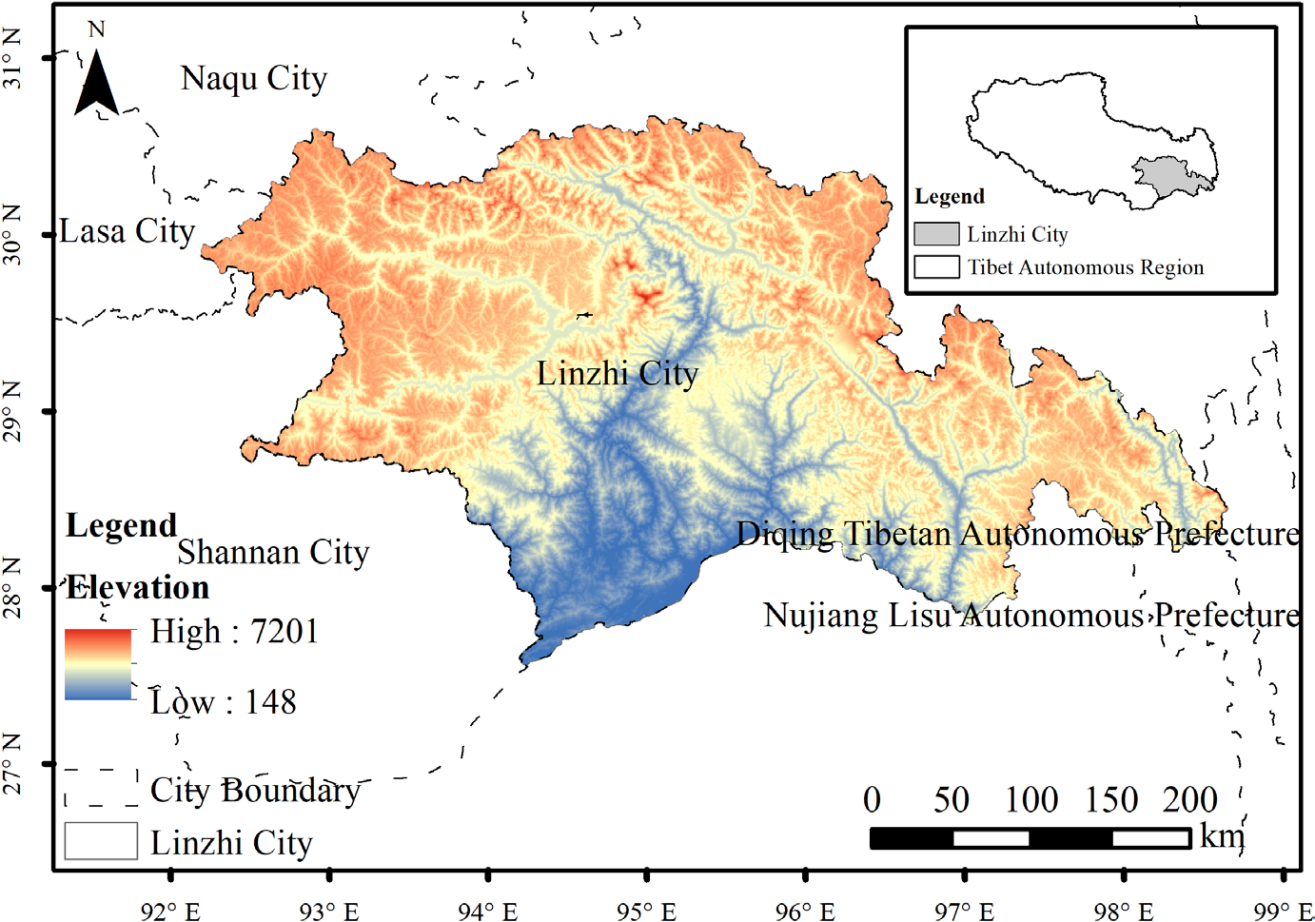
Geographical location of the study area.

The Tibet Autonomous Region serves as the primary wintering ground for the black‐necked crane, providing critical habitat and essential resources for the species (Cambridge UK 2001). Previous studies indicate that wintering populations are predominantly concentrated in the mid‐reaches of the Yarlung Tsangpo River basin, including its tributaries, the Lhasa River and Nianchu River basins, collectively known as the “One River, Two Rivers” region. Additionally, significant wintering populations have been documented in Nyingchi City, located in southeastern Tibet. A prefecture‐level administrative unit encompassing one urban district and six counties, spans 114,400 km^2^. Nyingchi City lies within the lower reaches of the Yarlung Zangbo River (27°33′02″–30°40′26″ N, 92°09′43″–98°18′30″ E). It is bordered by Nagchu City to the north, China–India and China–Myanmar international boundaries to the south, Chamdo City and Diqing Tibetan Autonomous Prefecture (Yunnan Province) to the east, and Shannan City and Lhasa City to the west (Figure 1). With an average elevation of 3100 m and a total area of approximately 114,400 km^2^, the region features a northwest‐to‐southeast topographic gradient. The warm and humid airflow from the Indian Ocean is blocked by the southeast terrain and forced to uplift, creating a humid subtropical climate characterized by mean annual temperatures ranging from 7°C to 16°C and an average annual precipitation of 1300 mm. Nyingchi City is also famous for its extensive lake and river systems, supporting wetlands and forest ecosystems.

### 2.2 Methods

MaxEnt and RF model are widely recognized as high‐performing SDMs (Mi, Huettmann, et al. 2017; Mi, Guo, et al. 2017). MaxEnt, based on ecological niche theory, utilizes the principle of maximum entropy to predict species distributions. It incorporates species occurrence records and known environmental variables as constraints. In the initial stage, we do not make biased assumptions about the unknown factors and assume that they have the same probability of occurrence, thereby identifying suitable habitat areas for target species (Regmi et al. 2018). RF, an ensemble machine learning algorithm based on decision trees, constructs multiple decision trees by randomly sampling datasets and feature subsets. The final prediction is determined by aggregating outputs from these trees, with high accuracy and robustness making it a popular choice for species distribution modeling (Mi, Huettmann, et al. 2017; Mi, Guo, et al. 2017). The data of this study were taken from the geospatial data cloud platform (https://www.gscloud.cn/) to obtain Landsat 8–9 OLI/TIRS C2 L2 image data in 2022, and the spatial resolution was set to 30 m. When selecting images, images with cloud cover < 10%, high imaging quality, and imaging time in the overwintering period of black‐necked cranes are preferred. Then, the image is preprocessed by ENVI 5.3, including radiometric calibration, atmospheric correction, and so on. According to the land use classification system of the Chinese Academy of Sciences and the characteristics of the study area, the study area is divided into six land types by using the supervised classification method. The study area is divided into six types of land: cultivated land, forest land, grassland, water area, construction land, and unused land.

In this study, two SDMs widely recognized by researchers were used to evaluate the habitat suitability of black‐necked cranes in Nyingchi City and predict their potential distribution areas. Based on species presence points and environmental variables, for MaxEnt, species presence records were combined with environmental variable raster layers within the MaxEnt software to generate habitat predictions. RF required preprocessing through ArcGIS software: Environmental variable values at both presence locations and pseudo‐absence points were extracted to create CSV‐formatted background datasets, which were subsequently analyzed using the Salford Predictive Modeler (SPM). The two models were tested by bootstrapping; all other settings remained at their default configurations. The use of R ecology is helpful to improve the reliability of the results. The datasets were partitioned into 70% training and 30% testing subsets, with this random partitioning process repeated 10 times to ensure statistical robustness.

The AUC is the area under the receiver operating characteristic (ROC) curve, which represents the probability that the model can correctly classify a randomly selected sample. AUC area was used to evaluate the performance of the model. This metric is independent of threshold settings and sample distribution (He and Zhou 2011; Wu et al. 2016). Theoretically, the AUC value ranges from 0 to 1, with higher values indicating greater accuracy of model prediction results (< 0.6 indicates that a model cannot differentiate preferred habitat from environment background data, and the model prediction fails; values between 0.6 and 0.7 are poor, between 0.7 and 0.8 they are fair, between 0.8 and 0.9 they are good, and > 0.9 they are excellent).

#### 2.2.1 Species Occurrence Data

Black‐necked cranes distribution data were retrieved from sources such as field surveys, websites, and literature. Model verification based on the species existence points in the literature is an important part of the species distribution model (SDM) evaluation. It needs to be combined with spatial statistical methods for comprehensive judgment, mainly including threshold‐dependent indicators, spatial explicit verification, etc. This monitoring effort was conducted under the framework of the 2024 Wintering Waterbird Synchronized Monitoring Program in the Tibet Autonomous Region, with systematic field observations carried out in Nyingchi City from 15 December 2023 to 15 January 2024. During field surveys, binocular high‐powered telescopes were utilized at each habitat of the black‐necked crane to promptly document the occurrence time, location, sex, and count of crane sightings. Furthermore, avian relics (such as feathers), tracks, and vocalizations were analyzed and recorded. GPS (Global Positioning System) technology was utilized to precisely determine the coordinates of each observation site. In cases where large flocks of black‐necked cranes were encountered, a high‐resolution digital camera was employed to promptly capture images, facilitating the enumeration and detailed documentation of the cranes.

The field survey adopted the method of combining sample points and sample lines. The sample lines were set up along the main roads, and the sample points were set up on the sample lines according to the actual distribution of black‐necked cranes. At the same time, the number, foraging position, and habitat characteristics of black‐necked cranes at each sample point were recorded. Field surveys were conducted during daylight hours using both walking and vehicle‐based transect methods. The direct count approach was adopted for crane monitoring due to the open terrain with sparse vegetation that allowed clear visibility. Survey routes specifically targeted black‐necked crane migration corridors and stopover sites, with route selection informed by local wildlife observers' field records and published survey data from previous studies.

In addition to the distribution points obtained through the field survey, this study accessed the Global Biodiversity Information Facility (GBIF, accessed on 1 May 2024) to obtain all historical records of black‐necked crane occurrences in Nyingchi City, Tibet Autonomous Region.

**FIGURE 2:**
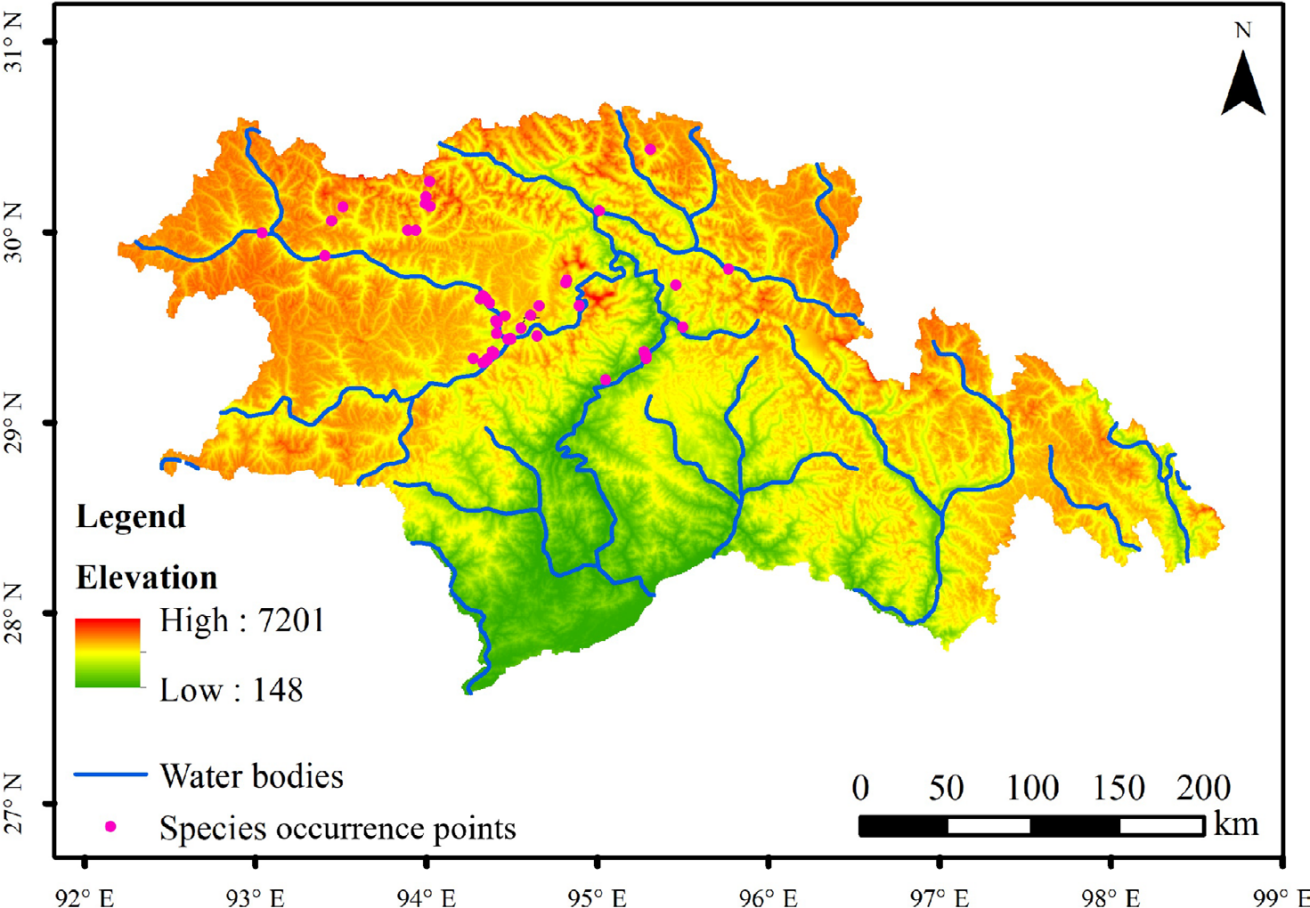
The existence point map of black‐necked crane in Linzhi City.

Historical GBIF records were cross‐validated with field survey data to eliminate outdated entries. To minimize potential bias and redundancy caused by clustering, spatial filtering of “presence points” was conducted to ensure that only one point was retained within each 1 km × 1 km raster grid. Records with coordinate errors (e.g., outside Nyingchi's geographic boundaries) were manually excluded. After removing duplicate points, a total of 52 “presence points” remained, which adequately met the requirements for model calculations. Thirty‐three sites were from field surveys, and nineteen sites were from online databases and published literature. The 52 points were obtained after screening (Figure 2). “Pseudo‐presence points” were generated using a random function within the study area, producing a total of 520 points (10 times the number of “presence points”) (Han et al. 2017). In this paper, the pseudo‐existence points are selected, which are generated by spatial perturbation of known existence points, and can be used to increase the stability of model training. Pseudo‐negative samples are randomly distributed. Black‐necked cranes have habitat exclusion, such as obvious exclusion in arid areas and areas with intensive human activities.

#### 2.2.2 Selection and Determination of Environmental Variables

Wildlife habitats must provide resources such as shelter, water, and food. The distribution of cranes is also limited by resources, so key factors such as distance from water, distance from roads, and climate variables (Bio1, Bio2) should be selected for habitat modeling. Crane habitat selection is not random; environmental variables influencing crane habitat choice primarily include water sources, food availability, and disturbance sources. Among these, food availability is the most critical factor affecting foraging site selection. Cultivated land serves as the primary foraging habitat for overwintering cranes in Tibet (He et al. 2011; Jia et al. 2019). Cranes predominantly select foraging sites near water sources, as wetland environments provide relatively safe foraging conditions (Li et al. 2009). Additionally, cranes frequently forage in farmlands surrounding residential areas, making them susceptible to human activities and disturbances from motorized vehicles (Cangjue et al. 2008) demonstrated that crane foraging sites exhibit significant spatial stability; when foraging site habitats remain relatively unchanged, the same site is re‐used across multiple wintering periods (Kong et al. 2011). Similarly, Qian et al., through the analysis of GPS locations of cranes wintering in alpine wetlands, confirmed this phenomenon (Qian et al. 2010). In terms of elevation, cranes are primarily distributed between 1900 and 5000 m, and they predominantly inhabit gentle slopes (< 30°) (National Forestry Bureau Central South Forestry Survey Planning Design Institute 2008; Liu et al. 2006).

In summary, most crane habitats are situated in areas characterized by flat terrain, open topography, and abundant food resources. Considering the life habits of cranes and based on the results of field surveys, four key indicators were selected for habitat suitability assessment: topography and geomorphology, foraging conditions, human disturbances, and climate conditions. Detailed information and sources of the environmental variables are provided in Table 1.

**TABLE 1:** Selected environment variable factor.

Variable	Code	Environmental variable	Data source website
Topography and geomorphology	alt	Altitude (m)	gebco.net/
	slp	Slope (°)	gebco.net/
	asp	Aspect (°)	gebco.net/
Foraging condition	d_wb	Distance from water body (m)	openstreetmap.org/
	d_r	Distance from road (m)	openstreetmap.org/
	d_b	Distance from building (m)	Resource Science and Data Platform (resdc.cn)
	vc	Vegetation cover (%)	—
Human disturbances	lut	Land‐use type	Resource Science and Data Platform (resdc.cn)
	d_al	Distance from agricultural land (m)	Resource Science and Data Platform (resdc.cn)
Climate conditions	Bio1	Mean annual temp. (°C)	worldclim.org/
	Bio2	Mean diurnal temp. range (°C)	worldclim.org/
	Bio3	(Bio2/Bioc7) Isothermal property ratio	worldclim.org/
	Bio4	Standard deviation of seasonal temp. variation	worldclim.org/
	Bio5	Max temp. warmest month (°C)	worldclim.org/
	Bio6	Min. temp. coldest month (°C)	worldclim.org/
	Bio7	Temperature annual range (°C)	worldclim.org/
	Bio8	Mean temperature wettest quarter (°C)	worldclim.org/
	Bio9	Mean temperature driest quarter (°C)	worldclim.org/
	Bio10	Mean temperature warmest quarter (°C)	worldclim.org/
	Bio11	Mean temperature coldest quarter (°C)	worldclim.org/
	Bio12	Annual precipitation (mm)	worldclim.org/
	Bio13	Precipitation wettest month (mm)	worldclim.org/
	Bio14	Precipitation driest month (mm)	worldclim.org/
	Bio15	Precipitation seasonality	worldclim.org/
	Bio16	Precipitation wettest quarter (mm)	worldclim.org/
	Bio17	Precipitation driest quarter (mm)	worldclim.org/
	Bio18	Precipitation warmest quarter (mm)	worldclim.org/
	Bio19	Precipitation of coldest quarter (mm)	worldclim.org/

*Note:* From the four key indicators of topography, foraging conditions, human disturbance, and climatic conditions, a total of 28 environmental variable factors were selected for habitat suitability evaluation.

(1) Three habitat factors, altitude (alt), slope (slp), and aspect (asp), were selected for topographic and geomorphological indicators. The main reasons for choosing GEBCO (General Bathymetric Chart of the Oceans) data over SRTM data for terrain analysis are as follows: GEBCO is an integrated global terrain model for both oceans and land, with a broader coverage range; GEBCO data has higher accuracy, integrating multiple data sources, including multibeam bathymetric data and depth predictions based on satellite gravity data; the GEBCO dataset is regularly updated, incorporating the latest bathymetric and satellite data. (2) Four habitat factors—distance to water bodies (d_wb), distance to roads (d_r), distance to buildings (d_b), and vegetation cover (vc)—were selected to assess foraging conditions. Among these, vegetation cover (vc) was derived from Sentinel satellite imagery. (3) Two habitat factors—land‐use type (lut) and distance to agricultural land (d_al) were selected to evaluate the level of human‐induced disturbances. (4) Climate conditions were divided into current and future climate data. Current Climate Data: The current climate data layer consists of global raster data generated through spatial interpolation of records from weather stations worldwide. It includes 19 bioclimatic variables sourced from the World Climate Database. Future Climate Data: Future climate projections were derived from the Coupled Model Intercomparison Project Phase 6 (CMIP6) shared socio‐economic pathways (SSPs) (Riahi et al. 2017). The time periods for future climate prediction are 2030s, 2050s, 2070s, and 2090s, respectively. Specifically, Shared Socioeco‐nomic Pathway 1–2.6 (SSP126) and Shared Socioeconomic Pathway 5–8.5 (SSP585) were selected as representative scenarios. SSP126 represents the “green, low‐carbon, and sustainable development” pathway, characterized by the lowest greenhouse gas emissions. SSP585 represents the “high‐carbon economic development” pathway, characterized by continuously increasing greenhouse gas emissions and concentrations. These two extreme climate scenarios were used to define threshold values for species distribution, providing a more effective means of assessing the impact of climate change on species' suitable habitats.

To ensure consistency in data accuracy and spatial compatibility, all datasets were uniformly projected to the WGS_1984_UTM_Zone_46N coordinate system, with a spatial resolution of 500 m.

**FIGURE 3:**
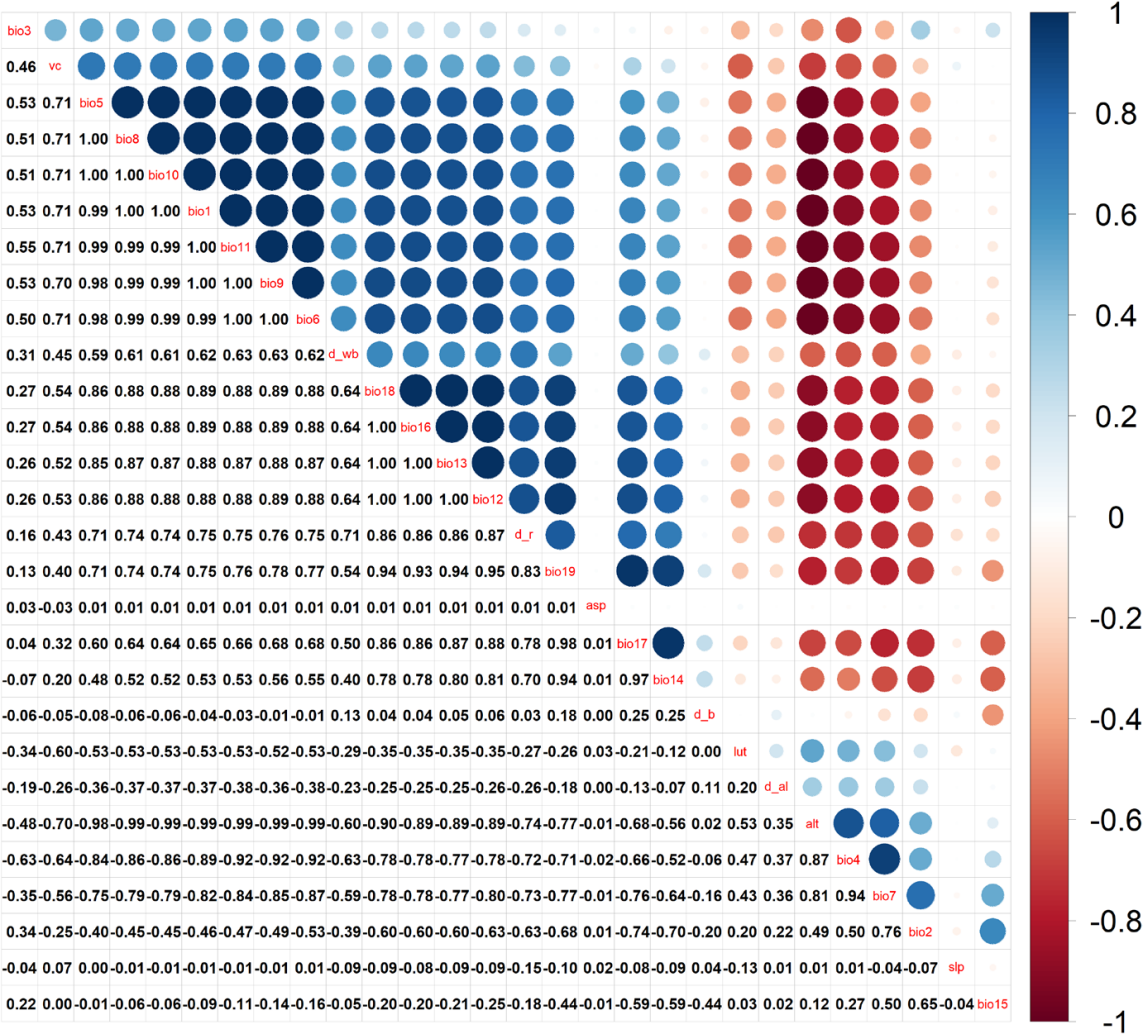
Heat map for correlation analysis of environmental factors. The darker the blue and red circles, the greater the correlation between environmental variables.

All environmental variables were imported into the model for analysis. However, due to the potential correlations between environmental variables, which can lead to overfitting, correlation analysis was conducted using the R package ENMTools (version 1.4.4) (Warren et al. 2021). This package does not rely on species distribution data and provides reliable results (Figure 3). The correlation plot was processed using the R package corrplot (version 0.92), and environmental variables with |R| ≥ 0.8 between the two factors were excluded. A Jackknife method was then applied for further screening, removing environmental variables with zero contribution. Ultimately, out of the original 28 environmental variables, 14 were retained: vc, asp, Bio2, Bio3, Bio5, Bio7, Bio14, Bio15, d_b, d_al, d_r, d_wb, lut, and slp.

## 3 Results

### 3.1 Model Evaluation

**FIGURE 4:**
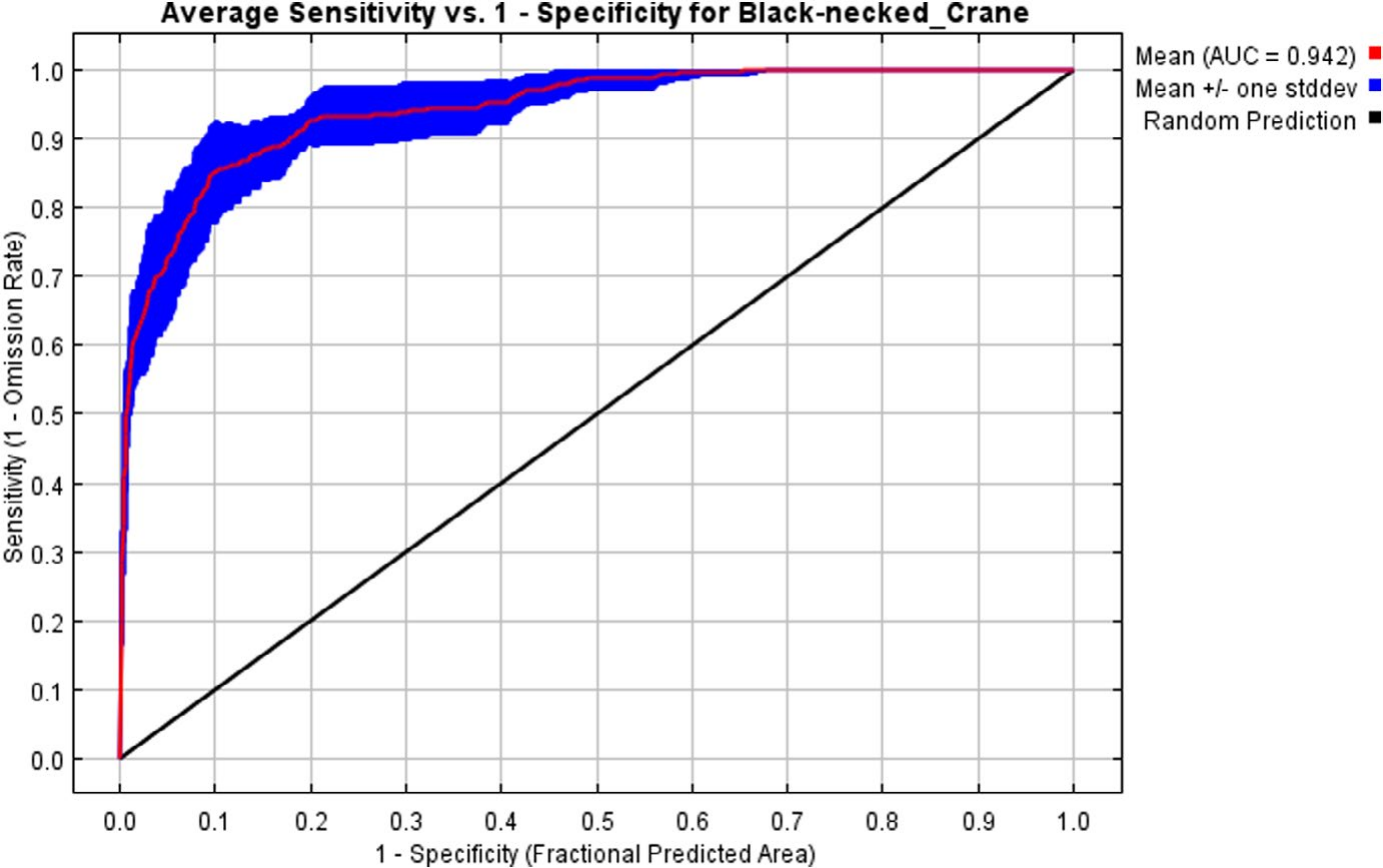
AUC results plot.

After using bootstrap, the simulation results of the two species distribution models show that the mean AUC values for both models tended to be stable (±0.001), effectively reducing random error caused by the selection of background points. The MaxEnt AUC value (0.942) was very high (> 0.9), as was the RF AUC value (0.945) (Figure 4). Both models demonstrated extremely high prediction accuracy, with AUC values exceeding the threshold for “excellent” performance and meeting the required precision for model application. While the MaxEnt exhibited slightly lower precision compared to the RF, the difference between the two was relatively small.

### 3.2 Analysis of Major Environmental Variables

The relative contributions of environmental variables to black‐necked crane distributions in Nyingchi City, derived from MaxEnt and RF, are summarized in Table 2. In the MaxEnt, land‐use type (lut) emerged as the predominant driver (30.6% contribution), followed by distance to buildings (d_b; 23.0%), distance to roads (d_r; 21.7%), and isothermality (Bio3; 5.1%). Conversely, the RF identified distance to water bodies (d_wb; 10.6%) as the most influential variable, with secondary contributions from maximum temperature of the warmest month (Bio5; 10.4%), mean diurnal temperature range (Bio2; 8.8%), and temperature annual range (Bio7; 8.5%).

**TABLE 2:** Retained environmental variables that contribute to the MaxEnt and RF models.

Code	Environmental variables	Environmental variables contribution (%)	
		MaxEnt	RF
vc	Vegetation cover	0.2	4.4
asp	Aspect	1.6	3.7
Bio2	Mean diurnal temperature range	0.1	8.8
Bio3	Isothermal property ratio	5.1	6.6
Bio5	Max temp. (warmest month)	4.3	10.4
Bio7	Temp. annual range	0.3	8.5
Bio14	Precipitation (driest month)	4.5	7.3
Bio15	Precipitation seasonality	2.9	7.5
d_b	Distance from building	23	7.2
d_al	Distance from agricultural land	0.4	5.5
d_r	Distance from road	21.7	8.4
d_wb	Distance from water body	3.8	10.6
lut	Land use type	30.7	3.2
slp	Slope	1.6	7.9

*Note:* The correlation analysis of environmental variables was carried out, and the Jackknife method was used for further screening. The variables with a zero contribution rate were eliminated, and 14 environmental variables were retained.

There are 12 environmental variable factors with an average contribution rate > 5% in both models, which are land‐use type (lut); distance from buildings (d_b), roads (d_r), water bodies (d_wb), and agricultural land (d_al); isothermal property ratio (Bio3), maximum temperature in the warmest month (Bio5), mean diurnal temperature range (Bio2), mean annual temperature range (Bio7), slope (slp), precipitation seasonality (Bio15), and driest month precipitation (Bio14). Among these, three variables—distance from buildings (d_b), 15.1%, 15.05%, 5.85% indicated that (d_b), (d_r), and (Bio3) contributed a lot, respectively, in both models. These values indicate that these three environmental variables contribute significantly to the model predictions and can be considered key factors for predicting black‐necked crane distribution.

#### 3.2.1 Distance From Buildings (d_b)

Between 2010 and 2020, Nyingchi City experienced substantial socioeconomic development, with its population increasing from 195,100 to 238,900 and fixed‐asset investment growing from CNY 6.517 billion to CNY 12.512 billion. This growth was concentrated in low‐elevation river valleys characterized by flat terrain, driving significant land‐use transitions: built‐up areas and croplands expanded, while grasslands, unused lands, and forest cover decreased correspondingly. The proliferation of built environments reflects accelerated urbanization and non‐agricultural economic activities, accompanied by intensifying human‐wildlife spatial competition. These anthropogenic pressures have fundamentally altered wintering black‐necked crane habitat selection strategies, compelling displacement from traditional foraging grounds to marginal habitats farther from human settlements.

#### 3.2.2 Distance From Roads (d_r)

Due to the region's rugged terrain, data sampling was limited to accessible areas. Consequently, most black‐necked crane presence points were clustered near roads. This led to a significant correlation between distance from roads and crane habitat suitability. However, roads generate noise, dust, and other disturbances, which increase the frequency of crane vigilance behaviors and extend their potentially disrupting their behavioral patterns (Xu et al. 2013). It also interferes with the behavior rhythm of black‐necked cranes.

#### 3.2.3 Isothermal Property Ratio (Bio3)

Bio3 represents the inter‐annual temperature stability, which significantly influences food resources and consequently, the habitat suitability for black‐necked cranes. During the wintering period, foraging is the primary activity of these cranes, and fluctuations in food resources directly affect their habitat suitability and potential distribution areas. The optimal isothermal range for black‐necked cranes is between 32 and 48. Their response to Bio3 shows that habitat suitability increases with higher inter‐annual temperature stability, reaching near‐maximum suitability at an isothermal value of 48.

### 3.3 Distribution Prediction Under Different Climate Scenarios

#### 3.3.1 Current Climate Suitability of Habitat for Black‐Necked Cranes

**FIGURE 5:**
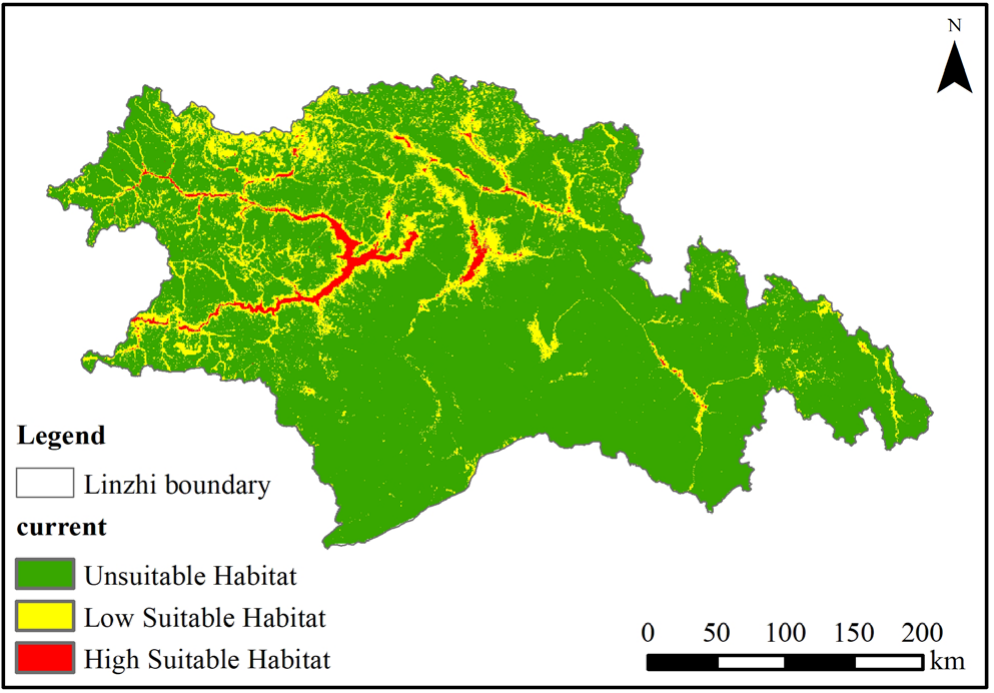
Habitat suitability map for black‐necked crane, Nyingchi City.

Based on existing research, this study employs the maximum TSS threshold (0.1657) and TPT balance threshold (0.0228) to classify black‐necked crane habitat suitability into three categories: unsuitable, low‐suitable, and high‐suitable habitats. Using the RF, which demonstrated the highest accuracy, we generated a habitat‐suitability map. The results, as shown in Figure 5, indicate that high‐suitable habitats are predominantly located within the Yarlung Tsangpo River basin, while low‐suitable habitats are distributed in other smaller watersheds. Overall, the suitable habitats for black‐necked cranes exhibit a fragmented distribution pattern.

#### 3.3.2 Potential Suitable Areas for Future Climate Black‐Necked Cranes

To simulate potentially suitable areas for black‐necked cranes, the atmospheric general circulation model (GCM) selected CMIP6. The periods of future climate prediction are 2021–2040, 2041–2060, 2061–2080, and 2081–2100; respectively, this study established four time scenarios (2030s, 2050s, 2070s, and 2090s) and two climate scenarios: SSP126, representing a green, low‐carbon, sustainable development pathway with minimal greenhouse gas emissions, and SSP585, representing a high‐carbon economic development pathway with continuously increasing greenhouse gas emissions and concentrations.

**FIGURE 6:**
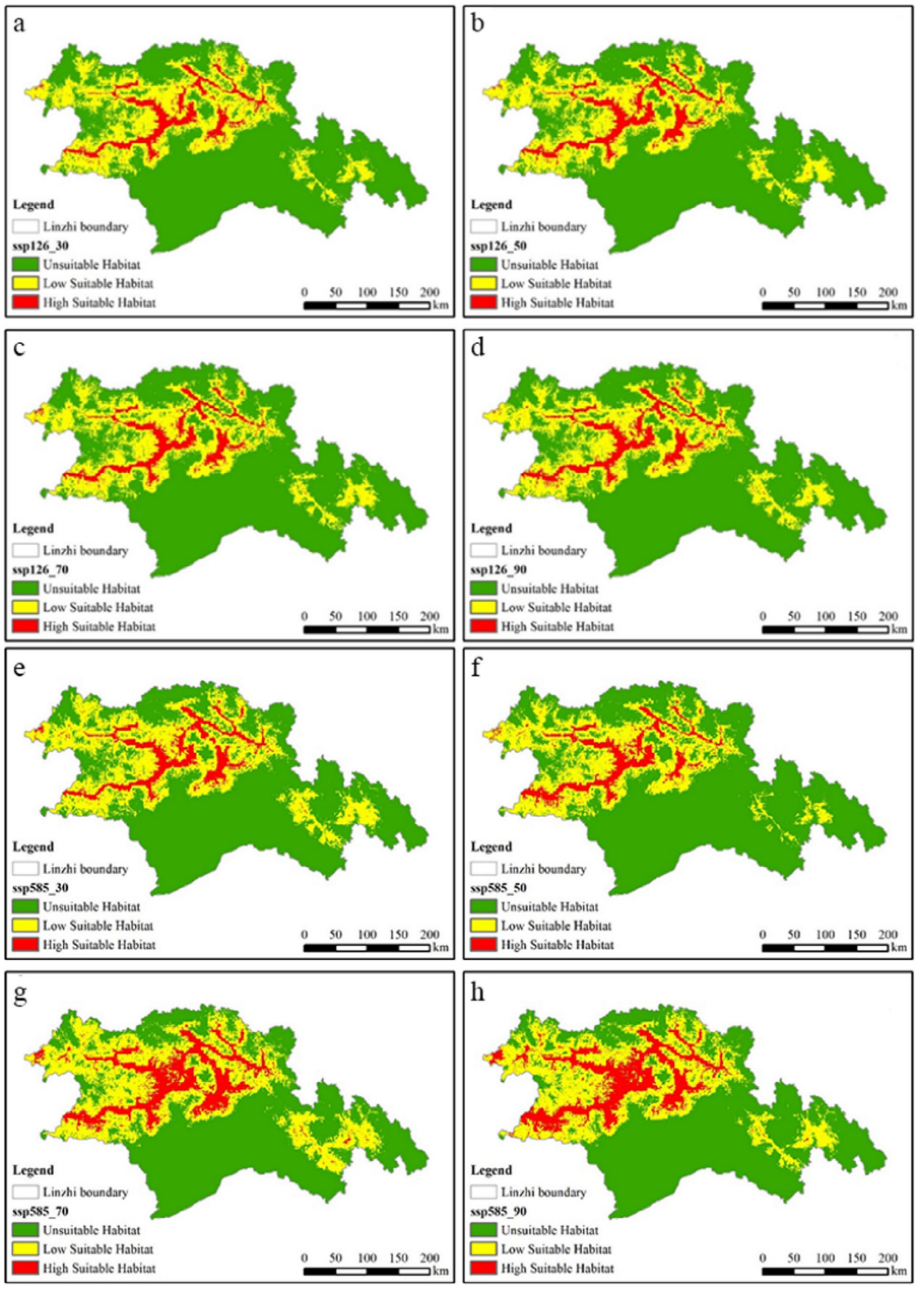
MaxEnt model to predict the suitable habitat distribution of black‐necked crane: (a) ssp126_30; (b) ssp126_50; (c) ssp126_70; (d) ssp126_90; (e) ssp585_30; (f) ssp585_50; (g) ssp585_70; (h) ssp585_90.

Figure 6 is the MaxEnt model prediction results of black‐necked crane habitat distribution under different climate scenarios. Under the SSP126 scenario, the overall pattern of habitat suitability remained relatively stable in the four periods (2030s, 2050s, 2070s, 2090s), and the conversion between habitat suitability grades (highly suitable, lowly suitable and unsuitable) was limited. On the contrary, under the SSP585 scenario, the total suitable habitat area and the highly suitable habitat area both peaked at the 2090s. The overall trend is gradually expanding suitable habitats, and moderately suitable habitats are gradually transformed into highly suitable habitats. These results suggest that the SSP126 scenario may represent unfavorable climatic conditions for this species compared to SSP585. The current distribution of black‐necked cranes is limited by the low temperature of the Qinghai‐Tibet Plateau (e.g., breeding cannot be completed in areas with an average annual temperature of < 5°C). Under the SSP585 climate model, there are phenomena such as the overall warming of the plateau, prolonged biological growth season, and increased precipitation. The habitat expansion of black‐necked cranes may be related to its heat resistance evolution.

**FIGURE 7:**
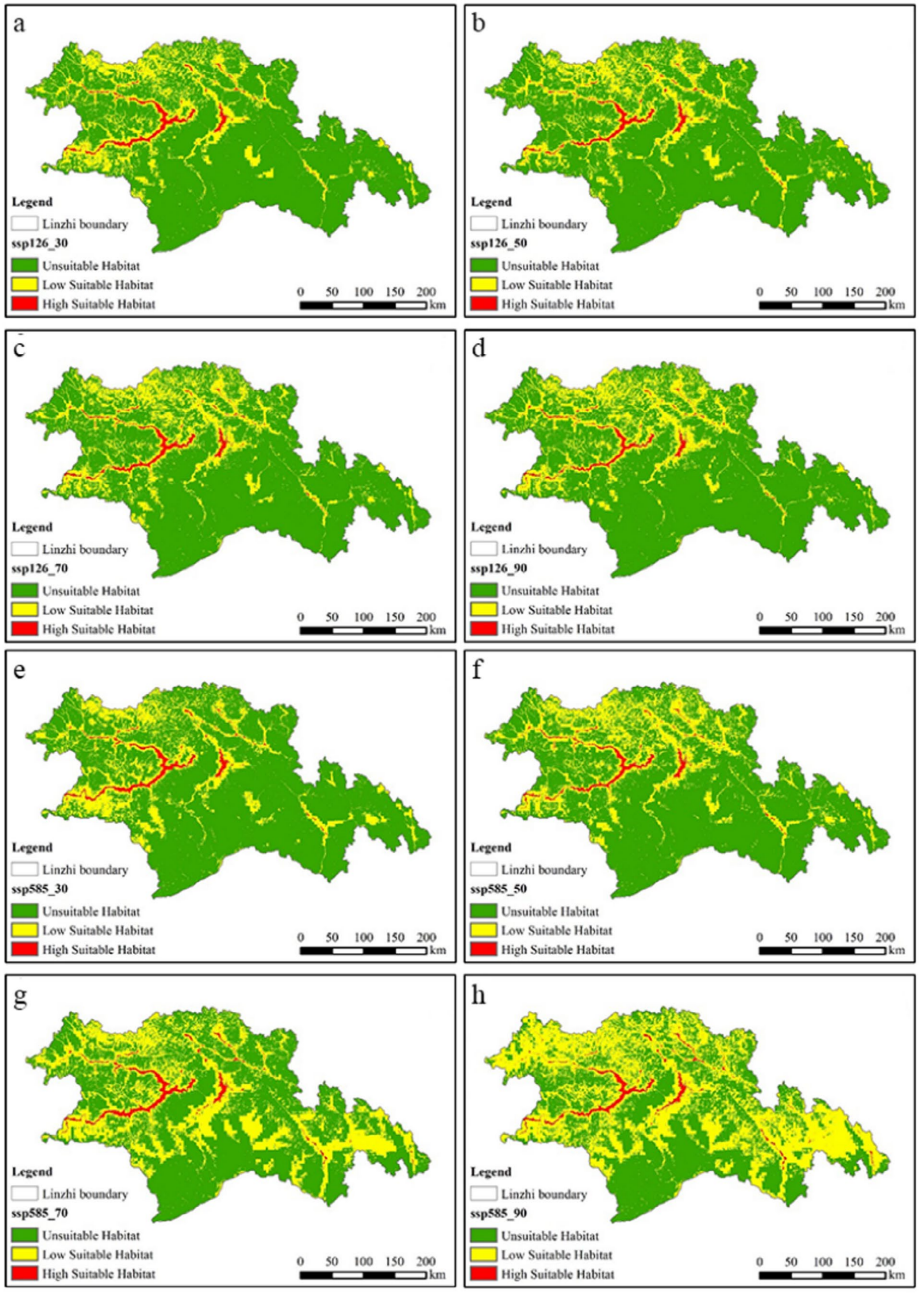
Random forest model to predict the suitable habitat distribution of black‐necked crane. (a) ssp126_30; (b) ssp126_50; (c) ssp126_70; (d) ssp126_90; (e) ssp585_30; (f) ssp585_50; (g) ssp585_70; (h) ssp585_90.

Figure 7 shows the prediction of the RF model on the distribution of black‐necked crane habitat under different climate scenarios. Under the SSP126 scenario, the highly suitable habitat area of cranes was the largest in the 2030s, and the total suitable habitat area was the largest in the 2090s, and the total suitable habitat area showed an overall upward trend. Under the SSP585 scenario, both the low suitable habitat area and the total suitable habitat area peaked in the 2090s, with the largest increase in the suitable habitat area between the 2070s and 2090s. In general, compared with the SSP126 scenario, the SSP585 scenario has more suitable habitats for black‐necked cranes.

## 4 Conclusions and Discussion

This study employed ME and RF models to reveal the spatial distribution and changes in crane suitable areas and potential suitable areas, and analyzed the future distribution of cranes under different climate change scenarios. The findings are as follows: (1) Both models demonstrated strong performance in predicting crane habitat suitability, with some differences. The RF model (AUC = 0.945) outperformed the ME model (AUC = 0.942). (2) By comparing the environmental variable factor contribution rates from both models, d_b, d_r, and bio3 emerged as key environmental variables influencing crane distribution predictions, with average contribution rates of 15.1%, 15.05%, and 5.85%, respectively. In this study, the habitat suitability of black‐necked crane in Nyingchi City was evaluated. (d_b), (d_r) and Bio3 are the three environmental factors with the highest contribution rate of the model. (d_b) and (d_r) are all indicators of foraging conditions, which emphasizes that foraging conditions are the key factors affecting the habitat suitability of black‐necked crane. (3) Under current climate scenarios, the suitable habitats for black‐necked cranes in Nyingchi City were generally fragmented within the T‐shaped river valley formed by the confluence of the Nyang rivers and the Yarlung Tsangpo River. (4) Under future climate scenarios, the distribution area of suitable habitats for black‐necked cranes in Nyingchi City is projected to increase, with the Yarlung Tsangpo River basin remaining the primary habitat area. The increased habitat suitability of black‐necked cranes under the SSP585 scenario found in this study is in stark contrast to the expectation that most high‐altitude species are threatened by climate warming (such as the reduction of the suitable area of snow leopard *Panthera unica* under SSP585) (Fabrice et al. 2022). However, a similar phenomenon has been reported in some wetland‐dependent birds: the arctic black goose ( *Branta bernicla* ) has expanded its breeding area due to warming tundra and prolonged growing season (Pere Pons 2020). The response of black‐necked cranes may be due to their high adaptability to hydrological changes, and existing models often underestimate the buffering capacity of wetland species to climate warming (such as snowmelt replenishment to maintain wetlands). Warming may increase the productivity of wetland invertebrates such as chironomid larvae, while black‐necked cranes have shown a certain degree of recipe plasticity, and the number and types of food they obtain have further increased.

This study shows that as a flagship species in plateau wetlands, the expansion of habitat suitability of black‐necked cranes under the SSP585 scenario reveals the atypical response pattern of high‐altitude species to climate change. The hydrological stability in the study area is very important for the survival of the population, and more targeted protection can be carried out by constructing ecological corridors and promoting “migratory bird‐friendly agriculture”. The main limitations are the uncertainty of the model. Specifically, there is a deviation of about 15% in the prediction of plateau precipitation by CMIP6 (David et al. 2021).

This study selected four primary indicators—topography and geomorphology, foraging conditions, human disturbances, and climate—at a macro level, encompassing a total of 28 environmental variables. Compared to previous studies, this research incorporated the unique life habits of black‐necked cranes in Tibet and field survey data to enhance the accuracy of model predictions. In addition to the environmental variables indicated by the results of this study, landscape patterns and other factors also have significant impacts on the distribution of the black‐necked crane. The high AUC values achieved in this study further facilitate a deeper understanding of the ecological niche characteristics of black‐necked cranes. In this study, the MaxEnt (ME) model was optimized by adjusting two parameters, RM (regularization multiplier) and FC (feature classes), to reduce spatial autocorrelation and thereby enhance the model's predictive accuracy. However, the Random Forest (RF) model was only used with default parameters. In the future, the RF model can be optimized by increasing the number of trees and the maximum depth of the trees, among other methods, to achieve more accurate prediction results. Simulation results from two models indicate that the total suitable habitat area for black‐necked cranes in future climate scenarios shows an increasing trend, indicating the strong emphasis China places on ecological protection efforts. Future wetland restoration and environmental protection measures will be effectively implemented, enhancing the habitat suitability of black‐necked cranes in future climates and their ability to adapt effectively to various future climate changes. The integration of multiple models represents the current dominant research trend, offering higher simulation precision and more accurate regional suitability assessments. By comparing the MaxEnt model and the RF model, this study significantly reduces spatial distribution uncertainty, precisely identifies the optimal distribution areas, and con‐tributes to the conservation of crane habitats. While food availability and human disturbance indices are critical for crane habitat selection, their quantification at a regional scale remains challenging due to data scarcity, Future studies should integrate satellite‐derived crop phenology data and spatially explicit human footprint indices. In response to the research findings, efforts to protect the black‐necked crane can be enhanced through measures such as strengthening the protection and restoration of wetland ecosystems and promoting ecological agricultural management practices. Seasonal habitat changes were partially captures through bioclimatic variables (e.g., Bio3 reflects temperature stability), but finer temporal resolution (e.g., monthly) could enhance model realism. Furthermore, future species distribution prediction studies should integrate multiple models, such as Biomod2, which is capable of constructing and evaluating species distribution models (SDMs). Biomod2 integrates various statistical and machine learning methods, such as generalized linear models (GLM) and support vector machines (SVM), to predict and analyze species geographic distribution under different environmental conditions. By evaluating models using multiple statistical indicators, such as TSS (True Skill Statistics) and Kappa coefficients, the accuracy and appropriateness of species distribution predictions can be significantly improved.

## Author Contributions

**Jiujiu Wu:** conceptualization (lead), data curation (lead), formal analysis (lead), investigation (equal), methodology (lead), resources (lead), software (equal), validation (lead), visualization (lead), writing – original draft (lead), writing – review and editing (lead). **Min Zheng:** investigation (equal), software (equal). **Zhongbin Wang:** funding acquisition (lead), project administration (lead), supervision (lead).

## Ethics Statement

The authors have nothing to report.

## Consent

The authors have nothing to report.

## Conflicts of Interest

The authors declare no conflicts of interest.

## Data Availability

Data are contained within the article.
